# Abschluss statt Ausschluss – die klinische Diagnosesicherung funktioneller Bewegungsstörungen

**DOI:** 10.1007/s00115-024-01613-9

**Published:** 2024-02-14

**Authors:** Anne Weissbach, Christina Bolte, Alexander Münchau

**Affiliations:** Institut für Systemische Motorikforschung, CBBM, Universität zu Lübeck, Marie-Curie-Straße Haus 66, 23562 Lübeck, Deutschland

**Keywords:** Ausschlussdiagnose, Klinische Charakteristika, Inkongruenz, Inkonsistenz, Diagnosekriterien, Diagnosis by exclusion, Clinical characteristics, Incongruence, Inconsistency, Diagnostic criteria

## Abstract

Funktionelle neurologische Bewegungsstörungen sind in der neurologischen Praxis häufig und führen zu einer hohen Beeinträchtigung und Chronifizierung. Betroffene erhalten meist erst mit langer Latenz eine Diagnose und häufig keine krankheitsspezifische Therapie. Eine Ursache dieser Verzögerung ist die häufig im Vorfeld durchgeführte umfangreiche apparative Diagnostik, die meist vorrangig dem Ausschluss anderer neurologischer Erkrankungen dient. Diese unauffällige Diagnostik wird dann häufig genutzt, um die funktionelle Bewegungsstörung als Ausschlussdiagnose zu kommunizieren. Patienten fällt es dadurch schwer, die Diagnose zu verstehen und anzunehmen. Dies ist besonders bedauerlich, da bei einem Großteil der Patienten die Diagnose sicher anhand der klinischen Charakteristika – Inkonsistenz und Inkongruenz – zu stellen ist. Die Erklärung der Symptome und die sich daraus ergebenden Therapieoptionen sollten zudem um patientengerechte Erläuterungen der pathophysiologischen Grundlagen der Erkrankung ergänzt werden. Die Patienten werden dadurch befähigt, die Diagnose einer funktionellen Bewegungsstörung ganzheitlich zu verstehen und zu akzeptieren. Dies kann wiederum die zum Teil jahrzehntelang andauernde Suche nach einer Diagnose zu einem Abschluss bringen, was den Weg in die Therapie ebnet. Die „Abschlussdiagnose“ wird damit zu einem Startpunkt der Behandlung und kann an sich bereits therapeutisch wirksam sein.

## Abstract (en)

Functional neurological movement disorders are common in neurological practice and lead to a high degree of impairment and chronification. Affected patients usually receive a diagnosis with considerable delay and often do not get disease-specific treatment. The reasons for this delay are related to extensive diagnostic measures to exclude other nonfunctional neurological diseases. As a consequence, functional movement disorders are typically communicated as diagnoses of exclusion, which makes it difficult for patients to understand and accept the diagnosis. This is particularly unfortunate, because in the majority of patients the diagnosis can be made with confidence based on clinical features, i.e., inconsistency and incongruence. The clarification of the symptoms and the resulting treatment options should be supplemented by patient-friendly explanations of the pathophysiological basis of the disease. In this way, patients are enabled to understand and accept the diagnosis. Moreover, it can put an end to the search for a diagnosis, which can sometimes take decades, and paves the way for treatment. Thus, the diagnosis by exclusion itself becomes the starting point for treatment and can itself have a therapeutic effect.

## Bedeutung einer „Abschlussdiagnose“

Funktionelle neurologische Störungen, von denen die funktionellen Bewegungsstörungen eine der größten Gruppen ausmachen, gehören zu den häufigsten neuropsychiatrischen Erkrankungen [1]. Bedauerlicherweise werden trotz der Häufigkeit viele Patienten nicht oder nur mit erheblicher Latenz diagnostiziert [2, 3]. Dies liegt unter anderem darin begründet, dass der apparativen Diagnostik häufig eine große Bedeutung in der Diagnosestellung zugeschrieben wird. So erhalten die meisten Patienten mit funktionellen Störungen wiederholt und ausgedehnt sowohl Bildgebungs- als auch elektrophysiologische und Liquor- bzw. Labordiagnostik. Der Normalbefund dieser apparativen Diagnostik wird nicht selten als Voraussetzung angesehen, um sich auf eine „sichere“ Ausschlussdiagnose festzulegen. Für die Patienten ist dies häufig unbefriedigend. Sie haben das Gefühl, über Diagnosen informiert worden zu sein, an denen sie nicht leiden. Ihre eigentliche Erkrankung wird ihnen aber nicht anhand diagnostischer Kriterien vermittelt. Vielen Patienten fällt es daher schwer, die Diagnose einer funktionellen Störung zu verstehen und zu akzeptieren. In dieser Folge werden meist weitere Kollegen unterschiedlicher Fachdisziplinen konsultiert, die meist erneut apparative Diagnostik veranlassen. Die fehlende bzw. verzögerte Diagnosestellung führt häufig zu einer Chronifizierung der Erkrankung und nicht selten einem Ausscheiden aus dem Beruf [4].

Der Wunsch einer erneuten Anordnung diagnostischer Maßnahmen seitens der Behandler ist dabei längst nicht die einzige Barriere zur Diagnose [5]. In einem systematischen Review zur Diagnosestellung funktioneller Störungen in der Primärversorgung wurden u. a. sowohl von Seiten der Patienten wie der Behandler das Kommunikationsverhalten während der Konsultation und die Dominanz biomedizinischer Krankheitsmodelle als relevante Faktoren identifiziert, außerdem interaktionale und situationale Barrieren. Die Hindernisse zu einer erfolgreichen Diagnosestellung sind vielfältig und facettenreich und machen deutlich, dass weitreichende und mehrstufige Maßnahmen erforderlich sind, um eine angemessene Versorgung zu gewährleisten [6].

Im Fokus dieses Artikels steht mit der Durchführung der klinischen Untersuchung und Diagnosestellung nur ein Bruchteil dieser Hindernisse. Damit soll der Artikel als Stütze dienen, die durch diagnostische Unsicherheit und biomedizinische Krankheitsmodelle bedingte Barriere aus dem Weg zu räumen, ist aber lediglich nur ein Baustein in einer Reihe von Strategien für eine bestmögliche zeitnahe Versorgung (darunter u. a. auch die frühe interdisziplinäre Zusammenarbeit und Arbeit in Netzwerken, der Umgang mit Vermeidungsverhalten und somatischer Krankheitsüberzeugung oder die Zusammenschau mit funktionellen Befunden andere Disziplinen betreffend).

Die Diagnose wird klinisch durch die Inkongruenz und Inkonsistenz der Symptome gestellt

Um den Teufelskreis aus diagnostischer Unsicherheit und Initiierung weiterer diagnostischer Maßnahmen zu durchbrechen, ist es wichtig, positive klinische Diagnosekriterien hervorzuheben, anhand derer man die Diagnose rein klinisch aufbauend auf Anamnese und neurologischer Untersuchung stellen und dem Patienten vermitteln kann. Dieses Vorgehen wird schon seit längerem empfohlen und ist ausführlich beschrieben worden (z. B. in [5]). Ein zentraler Baustein für den umfassenden Diagnoseprozess ist dabei die Verfügbarkeit validierter klinischer Zeichen, von denen wir eine Auswahl vorstellen möchten, die sich in unserer klinischen Erfahrung bewährt haben.

Obwohl die Anamneseerhebung und körperliche Untersuchung bei Patienten mit funktionellen Störungen häufig zeitaufwendig sind, reduzieren sie bei erfolgreicher Diagnosestellung und -vermittlung zusätzliche Konsultationen und apparative Diagnostik und sparen dadurch nicht nur Zeit und Kosten [7], sondern tragen einen erheblichen Teil zur Diagnoseakzeptanz und Behandlungseinleitung bei [5].

Im Kontext funktioneller Störungen wird berechtigt kritisiert, dass diese üblicherweise darüber definiert werden, was sie nicht sind. Ähnlich könnte auch die Diagnose einer funktionellen Störung rekonzeptualisiert werden: Sie ist nicht nur „keine Ausschlussdiagnose“, sondern eine „Abschlussdiagnose“, den Abschluss einer nicht selten langen, zirkulären diagnostischen Odyssee markierend.

## Psychische Faktoren bei funktionellen Bewegungsstörungen

Psychiatrische Komorbiditäten sind bei Patienten mit funktionellen Bewegungsstörungen häufig [8]. Die neurologische Konsultation ist durch ein Spannungsverhältnis zwischen Psychologisierung und Somatisierung gekennzeichnet, das es aufzulösen gilt: Während in der neurologischen Konsultation und klinischen Untersuchung selbst ein zu früher Verweis auf psychiatrische Komorbiditäten und psychische Belastungsfaktoren von den Patienten oftmals als bagatellisierend und abwertend wahrgenommen wird und die Wahrscheinlichkeit der Psychotherapieakzeptanz verringert [9–12], ist ein Ansprechen von Belastungsfaktoren jedoch unerlässlich. Denn gleichzeitig ist das Bewusstsein für die Rolle psychischer Faktoren bei der Entstehung und Aufrechterhaltung funktioneller Störungen von prognostischer Bedeutung [13, 14]. Eine ausführliche Elaboration psychiatrischer Komorbiditäten ist später Voraussetzung für die (integrative) psychotherapeutische Behandlung, wie sie in der Leitlinie zu funktionellen Körperstörungen eingehend beschrieben ist [15, 16], die eine integrative neuropsychiatrische Weiterversorgung empfiehlt.

Die „Abschlussdiagnose“ leitet den Start der weiteren Behandlung ein

Gerade im Hinblick auf die begrenzten zeitlichen Ressourcen im neurologischen Erstkontakt kann zunächst das Verdeutlichen der klinischen Zeichen im Vordergrund stehen, um die Diagnosestellung und -vermittlung optimal leisten zu können. Dieses Vorgehen soll keinesfalls die Nachrangigkeit psychosozialer Faktoren oder psychiatrischer Komorbiditäten suggerieren – Achtsamkeit für diese ist sowohl für das Verständnis der Diagnose als auch die Einleitung jedweder Behandlung von fundamentaler Bedeutung. Die „Abschlussdiagnose“ der funktionellen Bewegungsstörung markiert damit lediglich den Abschluss der neurologischen Diagnostik und leitet den Start der weiteren Behandlung ein, die eine ausführliche neuropsychiatrische Exploration beinhalten sollte.

## Inkongruenz und Inkonsistenz der Symptome

In der klinischen Untersuchung von Patienten mit funktionellen Störungen lassen sich alle verfügbaren Tests/Auffälligkeiten unter den zwei Hauptcharakteristika – Inkongruenz und Inkonsistenz – zusammenfassen [17]. In Zusammenschau aller Informationen aus der Anamnese (und Fremdanamnese) und der klinischen Untersuchung zeigen sich die Symptomzusammensetzung und -ausprägung (das klinische Muster) funktioneller Bewegungsstörungen inkongruent, also nicht in Übereinstimmung zum einen mit neuroanatomischen oder neurophysiologischen Gesetzmäßigkeiten und zum anderen dem Beschwerdebild nicht-funktioneller neurologischer Bewegungsstörungen. Letzteres lässt sich beispielsweise bei der Wechselinnervation beobachten, wenn es zu kurzzeitigen, ruckartigen Unterbrechungen bei der Kraftentfaltung kommt, was inkongruent zu einer nicht-funktionellen Parese ist, die zu einer gleichmäßigen Kraftreduzierung führt. Ersteres ist beobachtbar, wenn Patienten durch bestimmte Druckpunkte oder Bewegungen ihre Symptome in Abhängigkeit ihrer Erwartung/Vorstellung beeinflussen können. Weitere Beispiele sind in Tab. 1 zu finden.

**Tab. 1:** Inkongruente und inkonsistente Befunde funktioneller Bewegungsstörungen

		Spezifität (%)	Sensitivität (%)
*Zittern*			
Inkongruenz	*Entrainment*: Patienten imitieren in einer nichtbetroffenen Extremität einen vom Untersuchenden vorgegebenen Rhythmus. Das funktionelle Zittern nimmt diese Frequenz an	75–100	8,3–91
	*Amplitudenzunahme* des Zitterns bei 0,5 kg Gewichtsbelastung	92	22–33
	*Whack-a-Mole-Zeichen*: Bei passiver Fixierung des betroffenen Körperteils geht das Zittern in eine andere Region über	Keine Angaben	
Inkonsistenz	*Bewegungen der nicht betroffenen Extremität* (Fingertapping) führen zu einer Abnahme des Zitterns	73,3	72,7
	*Suggestion*: Durch das Aufsetzen einer Stimmgabel verringern oder verstärken sich die Symptome	87,9	41,7
*Gang- und Gleichgewichtsstörung*			
Inkongruenz	*Windmühlenzeichen*: Untersuchende ziehen Patienten kurzzeitig von hinten an den Schultern. Patienten zeigen ausfahrende, rudernde Bewegungen mit den Armen, halten aber gut das Gleichgewicht	Keine Angaben	
	*Funktioneller Romberg-Stehversuch*: Im Romberg-Stehversuch zeigen Patienten starke Auslenkungen mit dem Oberkörper und den Armen, können diese aber gut ausgleichen und kehren immer wieder in die Neutral-Null-Stellung zurück	Keine Angaben	
	*Fallneigung zur Unterstützung*: Patienten schwanken beim Gehen in die Richtung der Wand, Untersuchenden etc.	Keine Angaben	
Inkonsistenz	*Schnelles Gehen*/*Joggen* führt zu einer Abnahme der Gangstörung	Keine Angaben	
	*Veränderung der Phänomenologie* unter erschwerten Gangproben	Keine Angaben	
*Schwächen/Lähmungen*			
Inkongruenz	*„Give-way weakness“*: Plötzliches komplettes Sistieren der Kraft bei zuvor normaler Kraftentfaltung	97,8–100	44–85
	*Absinken ohne Pronation* im Armvorhalteversuch	93–95	61–100
	*Wechselinnervation*: Bei Kraftentfaltung Wechsel zwischen Anspannung und Entspannung	keine Angaben	
Inkonsistenz	*Hoover-Zeichen*: Hüftextension des Beines auf die Unterlage (gegen die Hand des Untersuchenden) nicht möglich. Unter Hüftflexion des nichtbetroffenen Beines gegen den Widerstand des Untersuchenden kommt es reflektorisch zu einer Hüftextension im zuvor gelähmten Bein	97,8–100	56–87
	*Abduktorzeichen*: Abduktion des 5. Fingers oder im Bein nicht möglich. Unter Abduktion des 5. Fingers der nicht betroffenen Hand bzw. Beines kommt es unbewusst zu einer Abduktion im zuvor gelähmten Finger/Bein	100	100
	*Inkonsistenz der Symptome im Liegen und Stehen*, z. B. keine Fußbewegung im Liegen möglich, aber Zehen- oder Hackengang gelingt	*Keine Angaben*	
*Fehlhaltungen*			
Inkongruenz	*Kokontraktion* antagonistischer und agonistischer Muskelgruppen	*100*	*30*
	*Fehlendes Babinski-II-Zeichen* beim funktionellen Spasmus hemifacialis: kein Anheben der Augenbraue bei gleichzeitigem Augenschluss	*70*	*100*
Inkonsistenz	*Passive Bewegung* verursacht Zunahme des Muskeltonus und Fehlhaltung	*Keine Angaben*	
	*Veränderung* der Schwere und Art der Fehlhaltung *bei Alltagstätigkeiten* (Entkleiden, Lagewechsel, Halten) meist außerhalb der neurologischen Untersuchung besser beobachtbar	*Keine Angaben*	

Die Tabelle zeigt nur eine Auswahl klinischer Zeichen der Inkonsistenz und Inkongruenz. Angaben zur Spezifität und Sensitivität aus [41]

Eine allgemeine breite Expertise im Bereich der neurologischen Bewegungsstörungen, wie sie z. B. in Spezialambulanzen zu finden ist, kann sehr hilfreich zur Feststellung dieser Inkongruenz sein [18].

Die Inkonsistenz der Symptome und klinischen Zeichen wird in der Variabilität der Schwere und Art deutlich. So sind Symptome und Zeichen meist mit Beginn der körperlichen Untersuchung am stärksten ausgeprägt und nehmen an Intensität ab, sobald die Patienten ihre Aufmerksamkeit von der betroffenen Körperregion bzw. den Symptomen weglenken. Dies wird häufig von den Patienten bereits in der Anamnese berichtet. Einige Patienten haben z. B. beim Ausüben von Hobbys deutlich weniger Symptome, da sie dabei meist unbewusst ihre Aufmerksamkeit weg von der eigentlichen Bewegungsausführung und hin zu äußeren (nicht körpereigenen) Stimuli lenken, z. B. gelingt das Gehen nicht, jedoch das Tanzen zu Musik. Die bewusste Verlagerung von Aufmerksamkeit fällt Patienten mit funktionellen Störungen allerdings in der klinischen Untersuchung und auf Aufforderung sehr schwer. So können unserer Erfahrung nach selbst ein einfacher Rhythmus durch Faustschluss oder Fußauftreten nicht fehlerfrei imitiert oder leichte motorische und kognitive Aufgaben (Fingerabzählen, Monatsnamen rückwärts aufsagen) nicht korrekt ausgeführt werden.

Die Inkonsistenz bezieht sich neben der Schwere der Symptome auch auf die Art, also deren phänomenologische Ausprägung, welche sich meist durch eine Erhöhung des Schwierigkeitsgrades während der Untersuchung (z. B. erschwerte Gangproben oder Handbewegungsaufgaben) verändert [19].

## Gemeinsamkeiten und Besonderheiten der Subgruppen funktioneller Bewegungsstörungen

Phänomenologisch lassen sich verschiedene Subgruppen funktioneller Bewegungsstörungen unterscheiden. Zittern, Schwäche/Lähmung, Fehlhaltungen und Gangstörungen zählen zu den am meisten berichteten Symptomen, die häufig in Kombination auftreten [20]. Frauen machen mehr als 70 % der Patienten aus [20]. Das mittlere Erkrankungsalter liegt bei 40 Jahren, wobei Patienten mit Fehlhaltungen (35 Jahre) und Schwäche/Lähmungen (36 Jahre) meist früher erkranken [20]. In vielen Fällen (37–80 %) treten die Symptome plötzlich und nach einem Trigger (z. B. physisches oder psychisches Trauma, Operation, andere Erkrankung, Schmerzen) auf [21–23]. Meist erinnern Patienten auch Jahrzehnte später noch das genaue Datum und Ereignis, bei dem erstmals Symptome auftraten. Das Triggerereignis steht dabei nicht in einem direkten kausalen Zusammenhang, hat für die Patienten aber eine entscheidende Bedeutung für die Entstehung ihrer Symptome [24].

Allen Subgruppen funktioneller Störungen ist gemeinsam, dass die Patienten Aufforderungen meist verlangsamt, vorsichtig und mit großer visueller Kontrolle und unter großer Anstrengung (Huffing-and-Puffing-Zeichen; [25]) ausführen. Viele haben das Bedürfnis, eine erhöhte Kontrolle über die betroffene Körperregion auszuüben, was dazu führt, dass komplexe Bewegungsabläufe, wie z. B. die posturale Stabilität oder das Gehen, nicht mehr flüssig-unbewusst ablaufen können.

Abhängig von der Phänomenologie der Bewegungsstörung lassen sich die Inkongruenz und Inkonsistenz mit unterschiedlichen Tests untersuchen, die in den folgenden Abschnitten und Tab. 1 detailliert beschrieben werden.

## Funktionelles Zittern

Eine der größten Gruppen funktioneller Bewegungsstörungen macht das funktionelle Zittern aus, welches typischerweise irregulär in Frequenz, Amplitude und Richtung ist [26]. Ein wichtiges Zeichen für die Inkongruenz stellt das Entrainment dar. Patienten imitieren dabei z. B. durch repetitiven Faustschluss oder Fußauftreten in einem nicht betroffenen Körperteil einen vom Untersuchenden vorgegebenen Rhythmus [27]. Dabei nimmt die betroffene Extremität diese vorgegebene Frequenz an. Nicht jedes funktionelle Zittern zeigt ein Entrainment und nimmt vielmehr stattdessen ab oder sistiert [28]. Diese Inkonsistenz des Zitterns ist auch bei der Ausführung anderer motorischer oder kognitiver Aufgaben zu verzeichnen (Tab. 1). Unter Gewichtsbelastung kommt es in Inkongruenz zu anderen nicht-funktionellen Tremores zu einer Zunahme der Amplitude [26]. Außerdem kann bei passiver Fixierung des zitternden Körperteils der Tremor manchmal auf eine andere zuvor nicht betroffene Körperregion übergehen (Whack-a-Mole-Zeichen; [29]).

## Funktionelle Gang- und Gleichgewichtsstörungen

Funktionelle Gangstörungen sind eine der häufigsten und gleichzeitig heterogensten Gruppen funktioneller Bewegungsstörungen. Trotz der Heterogenität können diese oftmals aufgrund ihres Gangbildes in grobe Kategorien eingeteilt werden. Eine Auflistung dieser ist in Tab. 2 zu finden.

In der klinischen Untersuchung lassen sich einige klassische Charakteristika herausuntersuchen, die den meisten funktionellen Gangstörungen gemein sind und eine Abgrenzung zu anderen nicht-funktionellen Gangstörungen erlauben [30]. So zeigen Patienten mit funktionellen Gangstörungen ein verlangsamtes und vorsichtiges Gehen, bei dem es zu einer starken visuellen Kontrolle des Gehens kommt [31]. Diese Verlangsamung und Unsicherheit beginnt typischerweise unmittelbar mit der Ganguntersuchung und liegt nicht bei der Untersuchung der Beine auf der Untersuchungsliege etc. vor. So können die meisten Patienten im Sitzen auf einem Bürostuhl diesen ohne Probleme mithilfe ihrer Beine korrekt fortbewegen [32]. Beim eigentlichen Gangvorgang kommt es dann aber meist zu einem unökonomischen Gehen, welches viel Kraft und Aufmerksamkeit in Anspruch nimmt. Dabei werden häufig ein oder auch beide Beine in eine Fehlhaltung gebracht (Innenrotation, Supination im Fuß, Beugen im Knie, Überkreuzen der Beine) und der Muskeltonus zeigt sich stark erhöht [31].

Meist ist die Gangstörung mit einer großen Angst, das Gleichgewicht zu verlieren und zu stürzen, vergesellschaftet, was neben der starken Verlangsamung auch zu einem breitbasigen und/oder schwankenden Gehen führt. Manchmal bewegen sich Patienten, als ob sie auf einer Eisfläche gehen würden („walking on ice“; [33]). Im Stehen mit geschlossenen Augen zeigen viele Patienten ein ausgeprägtes Schwanken des Oberkörpers, welches manchmal auch mit rudernden, ausfahrenden Bewegungen der Arme einhergeht (Windmühlenzeichen; [31]), bei dem es aber in aller Regel zu einem Zurückführen des Oberkörpers in die Mittellinie kommt. Eine Fallneigung erfolgt meist in die Richtung der Unterstützung und kann durch eine Aufmerksamkeitsverlagerung auf haptische Reize (z. B. Schreiben von Zahlen/Buchstaben auf den Rücken der Patienten) reduziert werden [33].

**Tab. 2:** Häufige Subtypen funktioneller Gangstörungen. (Adaptiert nach [30, 31, 43])

Gangbild	Charakteristika
Scherenartig	Häufiges Überkreuzen der Beine, Muskeltonus meist erhöht
Wie auf Eis	Patienten laufen, als ob sie auf einer Eisfläche gehen würden
Astasia-Abasia	Alleine Stehen oder Gehen nicht möglich, Festhalten an einem Gegenstand oder einer anderen Person
Mit Fehlhaltung	Ein- oder beidseitige Fehlhaltung von Bein oder Fuß, z. B. Innenrotation oder Supination im Bein/Fuß
Breitbasig	Gang mit anhaltend vergrößerter Schrittbreite, zusätzlich Schwanken und ausfahrenden Armbewegungen
Hinkend	Anhaltende einseitige oder deutlich asymmetrische Beugung im Kniegelenk mit reduzierter Standphase im betroffenen Bein
Schleifend	Betroffenes Bein zeigt meist Schwäche und erhöhten Muskeltonus und wird hinterhergezogen
Langsamer, vorsichtig	Patient läuft exzessiv langsam, Schwierigkeiten/Zögern beim Beginn
Unökonomisch	Patient läuft mit dauerhaft gebeugten Knien

Typisch für die meisten funktionellen Gangstörungen ist, dass diese in ihrer Schwere und Art sehr inkonsistent sind. Dies bedeutet z. B., dass die Schwere der Gangstörung durch eine Zunahme des Gehtempos abnimmt und sich das Gehen dann einem physiologischen Gangbild annähert. Dabei ist es von Vorteil, eine lange Strecke für die Ganguntersuchung zu nutzen (Korridor, im Freien). Außerdem sollte versucht werden, während des schnellen Gehens die Aufmerksamkeit der Patienten von der Ausführung des Gehens auf andere motorische Programme (gleichzeitiges Arbeiten mit den Händen) oder kognitive Aufgaben (Rechnen, Lesen) zu lenken. Dies kann zum Teil besonders effektiv auf einem Laufband gelingen. Von besonderer therapeutischer Bedeutung ist zudem das Integrieren möglicher Hobbys (z. B. Sportarten wie Ballspiele, Tanzen), bei denen der Fokus der Aufmerksamkeit nicht auf der Bewegung der Beine, sondern auf anderen Stimuli (visuell: Ball, akustisch: Musik) liegt und bei denen das Gehen (die Bewegung der Beine) unbewusst ablaufen kann.

## Funktionelle Schwächen/Lähmungen

Funktionelle Gangstörungen sind nicht selten durch Schwäche der Beine mitbegründet. Bedeutend für diese funktionelle Schwäche ist es, dass sie auf der Untersuchungsliege meist erheblich stärker imponiert als beim Stehen oder Gehen. So ist meist im Liegen gar keine Kraftentfaltung möglich, die Patienten können aber dennoch auf ihren Beinen stehen und bewerkstelligen einen selbstständigen Lagewechsel. Bei dem Versuch der Kraftentfaltung gegen Widerstand in der betroffenen Extremität kommt es häufig zu einem Wechsel aus An- und Entspannung, welches als eine Wechselinnervation spürbar ist. Nicht selten zeigt sich nach initial voller Kraftentfaltung ein plötzlicher totaler Kraftverlust („give-way weakness“; [34]). Typischerweise kommt es auch bei einer Schwäche der Arme zu keiner Pronation während des Absinkens [35].

Die klinischen Diagnosekriterien sollten Patienten während der Untersuchung erlebbar gemacht werden

Die zeitliche Inkonsistenz der Beschwerden kann zudem in klinischen Tests (Tab. 1, Hoover- [36], Abduktionszeichen [37]) herausuntersucht werden und sollte immer für die Patienten erlebbar gemacht werden. Dazu ist entscheidend, dass die Patienten realisieren, dass Kraftentfaltung unbewusst (durch Aufmerksamkeitsverlagerung weg von der betroffenen Extremität) erheblich besser gelingen kann, als wenn versucht wird, diese zu kontrollieren.

## Funktionelle Fehlhaltungen

Anders als funktionelle Schwächen sind funktionelle Fehlhaltungen meist mit einer erheblich gesteigerten muskulären Anspannung assoziiert. Speziell bei der passiven Bewegung der betroffenen Körperregion nimmt der Muskeltonus zu und kann als sehr schmerzhaft empfunden werden. Mehr als 90 % aller funktionellen Fehlhaltungen betreffen die Extremitäten (meist im Sinne einer verdrehten/fixierten Hand/Fuß; [38]), deutlich seltener sind Fehlhaltungen des Halses/Nackens und des Gesichts (verzogener Mundwinkel, Auge). Patienten mit funktioneller Fehlhaltung fällt es meist sehr schwer, Aufmerksamkeit von der Fehlhaltung weg zu verlagern. Man sollte daher auch Beobachtungen außerhalb der eigentlichen neurologischen Untersuchung (Entkleiden, Gestikulieren während der Anamnese, Auspacken von Krankenunterlagen etc.) in die Beurteilung einfließen lassen.

Fehlhaltungen der Beine im Liegen verändern sich meist in ihrer Ausprägung, sobald die Patienten gehen. Ähnlich ist dies bei Fehlhaltungen im Gesicht, die meist in Ruhe erheblich anders als während des Sprechens und Lachens ausgeprägt sind. Abhängig von der betroffenen Körperregion, in der die Fehlhaltung vorliegt, finden sich unterschiedliche Untersuchungstechniken (Tab. 1). Einige Patienten berichten von einer sofortigen Linderung der Fehlhaltung nach i.m. Injektion von Botulinumtoxin, welches nicht der physiologischen Latenz bis zum Einsetzten der Wirksamkeit des Medikaments entspricht [39]. Eine klassische Geste antagonistique ist nur selten zu beobachten, bei der es z. B. bei zervikalen Dystonien durch bloße Berührung zu einer Reduktion der Fehlhaltung kommt. Patienten mit funktionellen Fehlhaltungen benötigen meist sehr viel Kraft/Druck, um die Fehlhaltung eigenständig zu verändern [40].

## Therapeutische Konsequenz der Diagnosestellung

Bereits während der Erstvorstellung sollten den Patienten im Rahmen der klinisch-neurologische Untersuchung die positiven Diagnosekriterien veranschaulicht werden. Dazu eignen sich z. B. die oben beschriebenen und in Tab. 1 zu findenden Untersuchungstechniken, von denen viele mittlerweile validiert sind und eine gute Spezifität und Sensitivität aufweisen [41]. Auch zeigen diese Untersuchungstechniken oftmals die Reduktion der Symptome unter Aufmerksamkeitsverlagerung auf und bilden somit eine Möglichkeit der Herleitung verschiedener Behandlungsansätze, welche z. B. auf Techniken basieren, die sich Strategien zur Aufmerksamkeitslenkung zunutze machen. Wie in den Leitlinien zu funktionellen Körperstörungen empfohlen, sollte schon früh die Notwendigkeit interdisziplinärer neuropsychiatrischer und neurophysiotherapeutischer Strategien hervorgehoben werden. Auch für die Diagnosestellung an sich ist es notwendig, kombinierte diagnostische Ansätze zu verwenden, die auf mehreren Faktoren beruhen, so beispielsweise die klinischen Untersuchungstechniken in Zusammenschau mit der Anamnese.

Die Verdeutlichung der Symptome und die sich daraus ergebenden Therapieoptionen sollten zudem um patientengerechte Erläuterungen der pathophysiologischen Grundlagen der Erkrankung ergänzt werden. Dies führt dazu, dass Patienten die Diagnose der funktionellen Bewegungsstörung als solche verstehen und annehmen können. Dies kann dann die zum Teil jahrzehntelang andauernde Suche nach einer Diagnose zum Abschluss bringen und ebnet den Weg in die Therapie. Die „Abschlussdiagnose“ wird damit also vielmehr zum Startpunkt der Behandlung und kann damit selbst bereits therapeutisch wirksam sein [42].

## Fazit für die Praxis

- Die Diagnose einer funktionellen neurologischen Bewegungsstörung kann in vielen Fällen sicher anhand der klinischen Charakteristika gelingen.
- Apparative Diagnostik zum Ausschluss anderer Diagnosen ist bei den meisten Patienten mit funktionellen Störungen nicht erforderlich.
- Die klinischen Diagnosepfeiler sind die Inkonsistenz und Inkongruenz der Symptome.
- Während der neurologischen Untersuchung sollten den Patienten die klinischen Charakteristika verdeutlicht werden.
- Bereits im Erstkontakt sollte die Diagnose als funktionelle neurologische Bewegungsstörung benannt und für den Patienten verständlich und nachvollziehbar erklärt werden.
- Das Aufzeigen der Symptomreduktion unter Aufmerksamkeitsverlagerung kann eine Brücke zur weiterführenden Therapie sein (Physiotherapie, kognitives Aufmerksamkeitstraining).
